# Onion (*Allium cepa L*.) Organosulfur Compounds: From Traditional Use to Modern Pharmacological Insights

**DOI:** 10.1002/fsn3.71519

**Published:** 2026-02-05

**Authors:** Yuanyuan Tang, De Lv, Yijing Tao

**Affiliations:** ^1^ Department of Pharmacy Hospital of Chengdu University of Traditional Chinese Medicine Chengdu China; ^2^ Department of Endocrinology Hospital of Chengdu University of Traditional Chinese Medicine Chengdu China; ^3^ Department of Cardiology Changshu Hospital Affiliated to Soochow University, Changshu No. 1 People's Hospital Changshu China

**Keywords:** molecular mechanism, onion, organosulfur compounds, physiological function, purification extraction and, structure–activity relationship

## Abstract

Onion (*Allium cepa L*.), as one of the earliest vegetables cultivated by humans, has a medicinal value that can be traced back to The times of ancient Egypt, Greece, and Rome. In recent years, with the development of analytical techniques and molecular biology, the unique organic sulfides (organosulfur compounds, OSCs) in onions have attracted extensive attention from the scientific community. This article conducts a detailed analysis of the chemical structural characteristics of the main organic sulfides in onions, compares the advantages and disadvantages of extraction techniques such as steam distillation, organic solvent extraction, and supercritical CO_2_ extraction, and elaborates on their mechanisms of action in regulating lipid metabolism, exerting antibacterial and antitumor effects, alleviating diabetes, and mitigating asthma. The article finally discusses the application prospects of onion organic sulfides in the treatment of diseases, as well as the current technical challenges, providing a reference for the determination of future research directions.

## Graphical abstract

This review systematically examines the chemical diversity, extraction methods, and pharmacological activities of organosulfur compounds (OSCs) in onions. Key mechanisms include lipid metabolism regulation, anti‐bacterial and anti‐tumor effects, hypoglycemic activity, and anti‐asthmatic potential. The article highlights future directions for OSC‐based therapeutics amid current technical and clinical challenges.

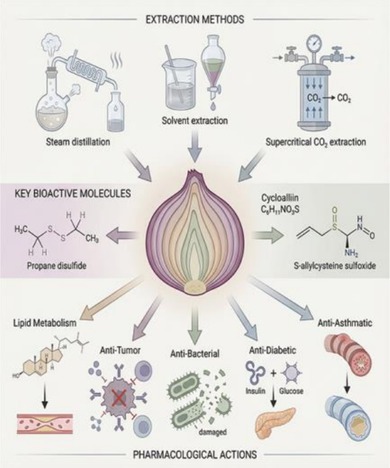

## 1 Introduction

Onion (*Allium cepa L*.), also known as jade onion, onion head, etc., is a plant of the genus Allium in the Liliaceae family (Yoon et al. 2024). The cultivation history of Onions can be traced back to Central Asia and the Mediterranean region around 3000 bc (Chadorshabi et al. 2022; Suleria et al. 2015). There are records about Onions in the murals of the ancient Egyptian pyramids (Kumar, Barbhai, Hasan, et al. 2022). In ancient Egypt, Greece, and Rome, Onions had already occupied an important position in folk medicine (Alzandi et al. 2022). The ancient Greek doctor Hippocrates once recommended using Onions to treat respiratory diseases, while the Roman scholar Pliny recorded 62 medicinal methods of Onions in his “Natural History” (Sharma et al. 2016). In China, the Compendium of Materia Medica records in detail the effects of Onions: “They are sweet and slightly pungent in taste, warm in nature, can unblock the five internal organs, reach all orifices, dispel cold and dampness, and ward off evil (Kumar, Barbhai, Hasan, Dhumal, et al. 2022).”

Onion, as a traditional plant that can be used both as medicine and food, has a relatively rich content of organic sulfur compounds (OSCs), which mainly exist in the vacuoles of the bulb (Muscolo et al. 2020; Qian et al. 2022). When the cell structure is damaged, allinase in the vacuole rapidly converts the odorless S‐alkane (ene)‐L‐cysteine sulfoxide into biologically active thiosulfonates, sulfides, and other compounds (Asemani et al. 2019; Kamata et al. 2016). This defense mechanism is also the chemical basis for the pungent smell and medicinal value of Onions.

Over the past two decades, the number of research papers on onion organic sulfides indexed in the PubMed database has increased nearly fivefold. The research focus has gradually shifted from the initial chemical composition analysis to the exploration of the mechanism of action and clinical application (Shang et al. 2019). At present, Japan, Germany, and the United States are in the leading position in this field. Although China's onion production accounts for nearly 40% of the global total, the development of deep processing and high value‐added products lags relatively behind. With the rising incidence of chronic diseases and the prominent issue of the side effects of chemical drugs, the development of onion organic sulfides, a natural drug resource, for the treatment of chronic diseases holds significant social and economic value (Sharma et al. 2017). The literature reviewed in this article is sourced from the Web of Science Core Collection, PubMed, and Scopus databases from 2000 to 2025, with a focus on the composition, extraction methods, and pharmacological effects of organic sulfides contained in Onions.

## 2 Classification and Characteristics of Organic Sulfides in Onions

The primary active constituent of onions is onion essential oil, a clear orange‐yellow volatile oil (Taghavi et al. 2022). Over 60 sulfur‐containing compounds have been identified in this oil (Kocić‐Tanackov et al. 2012). Based on their chemical structures, these compounds can be classified into three main categories (Marefati et al. 2021; Abrante‐Pascual et al. 2024; Cantrell et al. 2020). The first category comprises thiosulfinates, including propanethial‐S‐oxide, 1‐propenyl thiosulfinate, and methyl thiosulfinate. These compounds are highly reactive and serve as the primary contributors to the characteristic pungent odor of onions, as well as the foundation for numerous biological activities. The second category includes sulfides and disulfides, with representative compounds such as dipropyl disulfide, diallyl disulfide, and methyl allyl disulfide. The third category consists of cysteine derivatives, such as S‐allyl‐L‐cysteine sulfoxide (alliin), S‐methyl‐L‐cysteine sulfoxide, and cycloalliin. These derivatives are relatively stable and function as key flavor precursors in onions. Table 1 provides a systematic summary of the major OSCs in onions, including their classification, chemical names, molecular formulas, and principal biological activities and properties.

**TABLE 1:** The main organic sulfides found in onions and their biological activities and properties.

Category	Compound name	Chemical formula	Primary biological activity and properties	Reference(s)
Thiosulfinates	Propanethial‐S‐oxide	CH_3_CH_2_CH = S = O	The primary direct source of onion's pungent odor and lachrymatory (tear‐inducing) effect; possesses activities such as antibacterial and antiplatelet aggregation	Taghavi et al. (2022), Kocić‐Tanackov et al. (2012), Cantrell et al. (2020)
	1‐Propenyl thiosulfinate	CH_2_ = CHCH_2_‐S (O)‐S‐CH_3_	Highly chemically reactive, serving as the core material basis for many biological activities; can be converted into various sulfides in the body	Kocić‐Tanackov et al. (2012), Marefati et al. (2021)
	Methyl thiosulfinate	CH_3_‐S (O)‐S‐CH_3_	Chemically reactive, exhibits significant antibacterial and anticancer activity	Kocić‐Tanackov et al. (2012), Cantrell et al. (2020)
Sulfides and Disulfides	Dipropyl disulfide	(CH_3_CH_2_ CH_2_)_2_S_2_ or C_6_H_14_S_2_	The most abundant sulfur‐containing compound in onions, accounting for 80%–93% of total sulfur content; possesses physiological activities such as antioxidant, anticancer, and lipid‐lowering effects	Kocić‐Tanackov et al. (2012), Cantrell et al. (2020)
	Diallyl disulfide	(CH_2_ = CHCH_2_)_2_S_2_ or C_6_ H_10_S_2_	Low content (< 1%), but contributes to the main aroma of onions; possesses potent antibacterial, anticancer, and blood glucose‐lowering activities	Kocić‐Tanackov et al. (2012), Cantrell et al. (2020), Shala et al. (2023)
	Methyl allyl disulfide	CH_3_‐S‐S‐CH_2_CH = CH_2_ or C_4_H_8_S_2_	Exhibits various biological activities including anticancer and antibacterial effects; is an important flavor component in onion essential oil	Kocić‐Tanackov et al. (2012), Cantrell et al. (2020)
Cysteine derivatives	S‐Allyl‐L‐cysteine sulfoxide (Alliin)	C_6_H_11_NO_3_S	A precursor to thiosulfinates (e.g., 1‐Propenyl thiosulfinate); converted by the enzyme alliinase when onions are cut; possesses activities like hypoglycemic and antioxidant effects.	Kocić‐Tanackov et al. (2012), Subramanian et al. (2020), Yang et al. (2018)
	S‐Methyl‐L‐cysteine sulfoxide	C_4_H_9_NO_2_S	Similarly, a precursor to flavor compounds like methyl thiosulfinate; also exhibits hypoglycemic and antioxidant properties	Kocić‐Tanackov et al. (2012), Castro et al. (2021)
	Cycloalliin	C_6_H_11_NO_3_S	A natural sulfur‐containing amino acid in onions, stable and nonpungent; possesses physiological activities such as hypoglycemic and cholesterol‐lowering effects	Yanagita et al. (2003), Ichikawa et al. (2006)

## 3 The Extraction Method of Organic Sulfides in Onions

**FIGURE 1:**
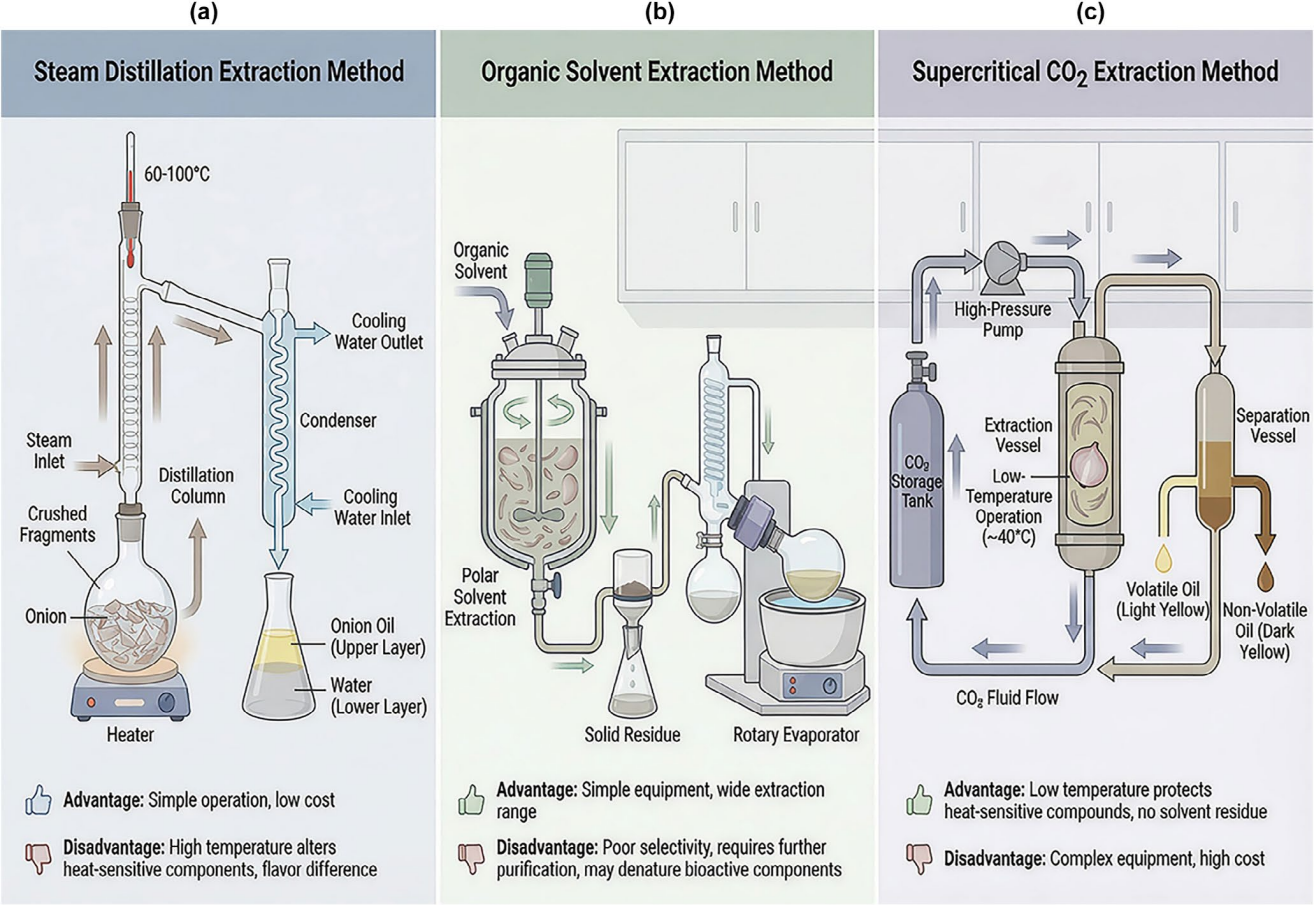
Comparison of three organic sulfide extraction methods. This illustration presents three methods for the extraction of onion essential oil. (a) Steam distillation method: Involves crushing fresh or dried onions, then conducting steam distillation under atmospheric or reduced pressure, followed by oil–water separation to obtain onion essential oil. (b) Organic solvent extraction method: Starts with crushing raw onions, then uses solvent immersion for extraction. After that, solvent evaporation and recovery are carried out, and the resulting crude extract requires further purification. (c) Supercritical CO_2_ extraction method: Begins by crushing fresh or dried onions, then performs supercritical CO_2_ fluid extraction. Subsequently, pressure reduction in a separation vessel is done to obtain high‐quality onion essential oil.

Most organic sulfides are present in onion essential oil. Traditional methods for the extraction of onion oil include atmospheric steam distillation, solvent extraction, and supercritical CO_2_ extraction. Figure 1 illustrates a comparison of the essential principles, equipment, and process flows of three primary methods for extracting organic sulfide from onions.

### 3.1 Steam Distillation Extraction Method

This is the most commonly used method for extracting the volatile oil (onion essential oil) from Onions (Wang et al. 2018). This method utilizes Dalton's law of partial pressure to separate components that are insoluble in water. Usually, fresh or dried scallions are crushed before distillation, and then steam is introduced under normal or reduced pressure to distill the onion essential oil at a temperature lower than its boiling point. This is mainly to obtain the more volatile essential oil in Onions. This method is simple to operate and requires less investment, but its disadvantages are that the distillation time is long, the oil yield is low, and the temperature during the steam distillation process is high, which causes some heat‐sensitive substances to change. This is extremely unfavorable for studying the effective components in the extract, especially certain biologically active components in the volatile oil. Therefore, the flavor of the extracted onion essential oil is somewhat different from that of raw scallions (Takahashi and Shibamoto 2008).

### 3.2 Organic Solvent Extraction Method

Solvent extraction method has the characteristics of simple equipment, low investment and wide extraction range (Ciardi et al. 2021). The principle of solvent extraction: By taking advantage of the differences in solubility and polarity of different substances in solvents, they become less likely to dissolve each other, thereby achieving the purpose of separation and purification (Trigueros et al. 2024). Based on the principle of like dissolves like, solvents of different polarities were selected to extract the effective components from Onions. However, due to the similar polarities of the effective components and the poor selectivity of organic solvents, the leaching method cannot only obtain nonpolar lipid components but also extract a large amount of polar substances. Single leaching cannot obtain high‐purity target products. Generally, further separation and purification are required. Meanwhile, organic solvents can easily destroy proteins, hormones, and enzymes in raw materials, causing denaturation and inactivation of bioactive substances, etc. (Srivastava and Balakrishnan 2022).

### 3.3 Supercritical CO_2_ Extraction Method

Supercritical CO_2_ extraction can be operated at relatively low temperatures, thus resulting in less decomposition loss of heat‐sensitive compounds. It can obtain high‐quality extracts without leaving any residual solvents. However, the equipment used is complex, the investment is large, and the operating conditions are harsh. Sometimes, the extraction effect is not very ideal (Tolcha et al. 2020). Using CO_2_ as a supercritical fluid, the essential oil from Onions is extracted. Then, the CO_2_ fluid carrying the essential oil undergoes a phase transformation from a supercritical state or liquid to a gas state to release the essential oil it carries. This method can simultaneously obtain volatile and nonvolatile onion essential oils (Putnik et al. 2019).

## 4 Biological Functions of Organic Sulfides in Onions

OSCs derived from onions have a wide range of biological properties, which involve lipid metabolism regulation, antibacterial effects, antitumor properties, hypoglycemic activity, and anti‐asthmatic potential. The following subsections provide an in‐depth account of the pharmacological actions and fundamental mechanisms of organic sulfides in onions.

### 4.1 Onion‐Derived Compounds in Regulation of Lipid Metabolism

Hyperlipidemia is a metabolic disorder characterized by elevated levels of lipids in the blood and represents one of the primary risk factors for cardiovascular diseases. The principal clinical consequence of hyperlipidemia is atherosclerosis, which results from the accumulation of lipids within vascular walls.

Li et al. (2021) investigated the lipid‐lowering effects of onion extract in hyperlipidemic rats and demonstrated that onion extract exerts an auxiliary hypolipidemic effect in Sprague–Dawley (SD) rats. Han et al. (2002) conducted an in vitro study using human hepatocytes to examine the influence of sulfur‐containing compounds in onions on lipid metabolism, particularly focusing on apolipoprotein B100 secretion. Their findings indicate that S‐propyl cysteine significantly reduces apolipoprotein B100 secretion, thereby inhibiting lipoprotein synthesis and secretion. (Yanagita et al. 2003) explored the impact of cycloalliin on lipid metabolism in SD rats and reported that cycloalliin derivatives modulate hepatic lipid synthesis and secretion, leading to reduced cholesterol levels. Castro et al. (2021) confirmed that S‐methylcysteine sulfoxide, a bioactive compound in onions, exhibits both hypoglycemic and hypolipidemic effects in alloxan‐induced diabetic mice. Furthermore, Ichikawa et al. (2006) demonstrated that sulfur‐containing amino acids—specifically S‐methylcysteine sulfoxide and S‐allylcysteine sulfoxide—can mitigate cholesterol elevation induced by high‐fat and heat‐sensitive diets.

**FIGURE 2:**
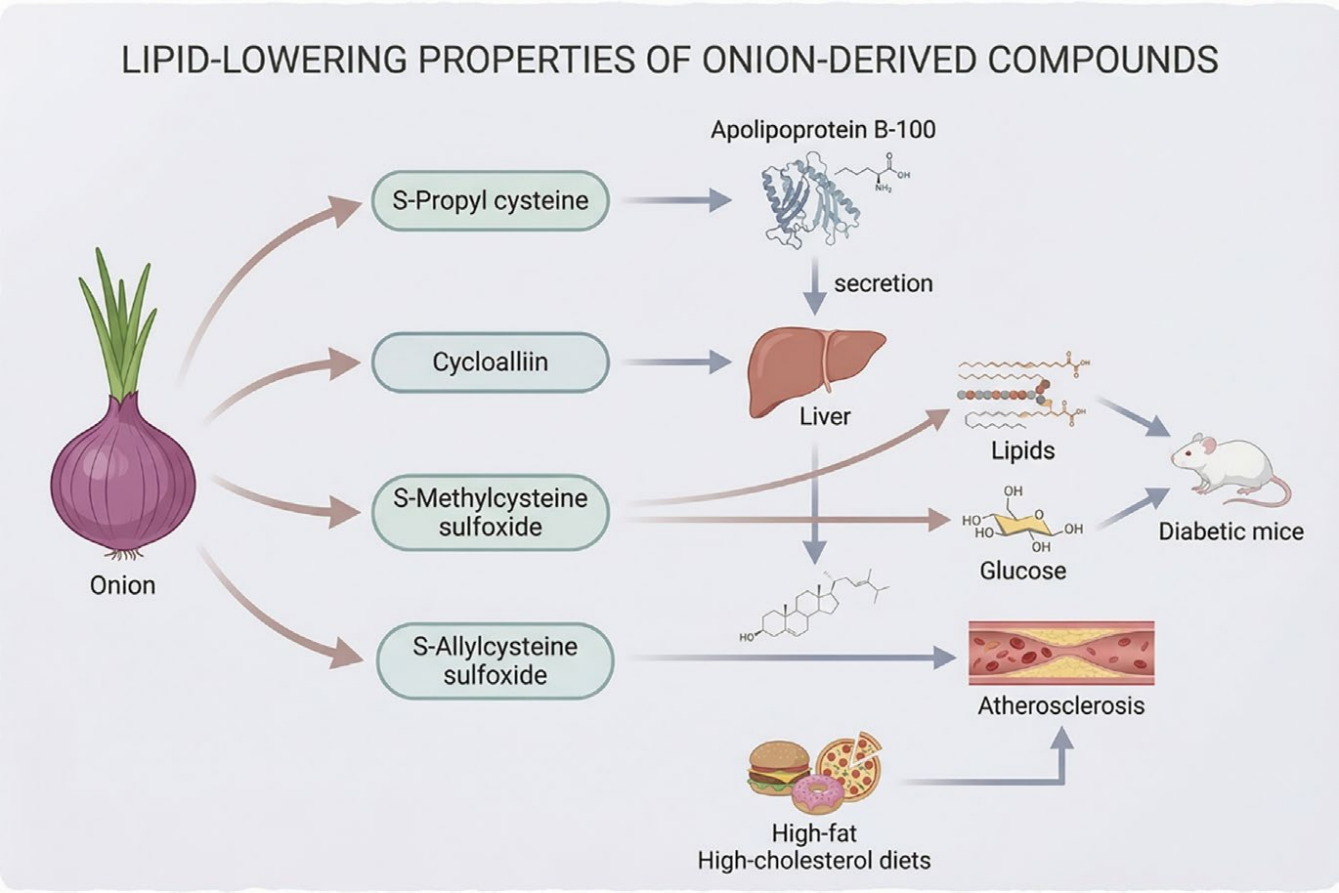
Lipid‐lowering properties of organic sulfides in onions. This image depicts the lipid‐lowering properties of onion‐derived compounds. S‐propyl cysteine significantly reduces apolipoprotein B100 secretion; cycloalliin derivatives modulate hepatic lipid synthesis and secretion, contributing to lower cholesterol levels; and S‐methylcysteine sulfoxide and S‐allylcysteine sulfoxide demonstrate potential in cholesterol reduction.

Overall, these findings show that sulfur‐containing flavor precursors in onions, such as S‐methylcysteine sulfoxide, which are enzymatically converted into volatile thiosulfinates responsible for onion flavor and biological activity, have significant lipid‐lowering properties, as shown in Figure 2.

### 4.2 Anti‐Bacterial Properties of Onion Sulfur Compound

Allicin compounds in fresh onion juice have a moderate bactericidal effect (Kong et al. 2024). Allicin and disulfide compounds can react with compounds similar to certain amino acids (e.g., cysteine) preventing them from binding to proteins. This reaction can inhibit bacterial reproduction (Hai et al. 2025). When Kim et al. (2018) conducted a study on the antibacterial activity of Onions, they found that sulfur‐containing compounds could effectively inhibit both Gram‐positive and Gram‐negative bacteria. At the same time, they believed that the sulfur substances in Onions also had a selective inhibitory effect on bacteria in the digestive tract. Lazarević et al. (2011) conducted a comparative study on 12 Allium species, including Onions, and found that sulfur compounds in Onions had the strongest antibacterial activity.

**FIGURE 3:**
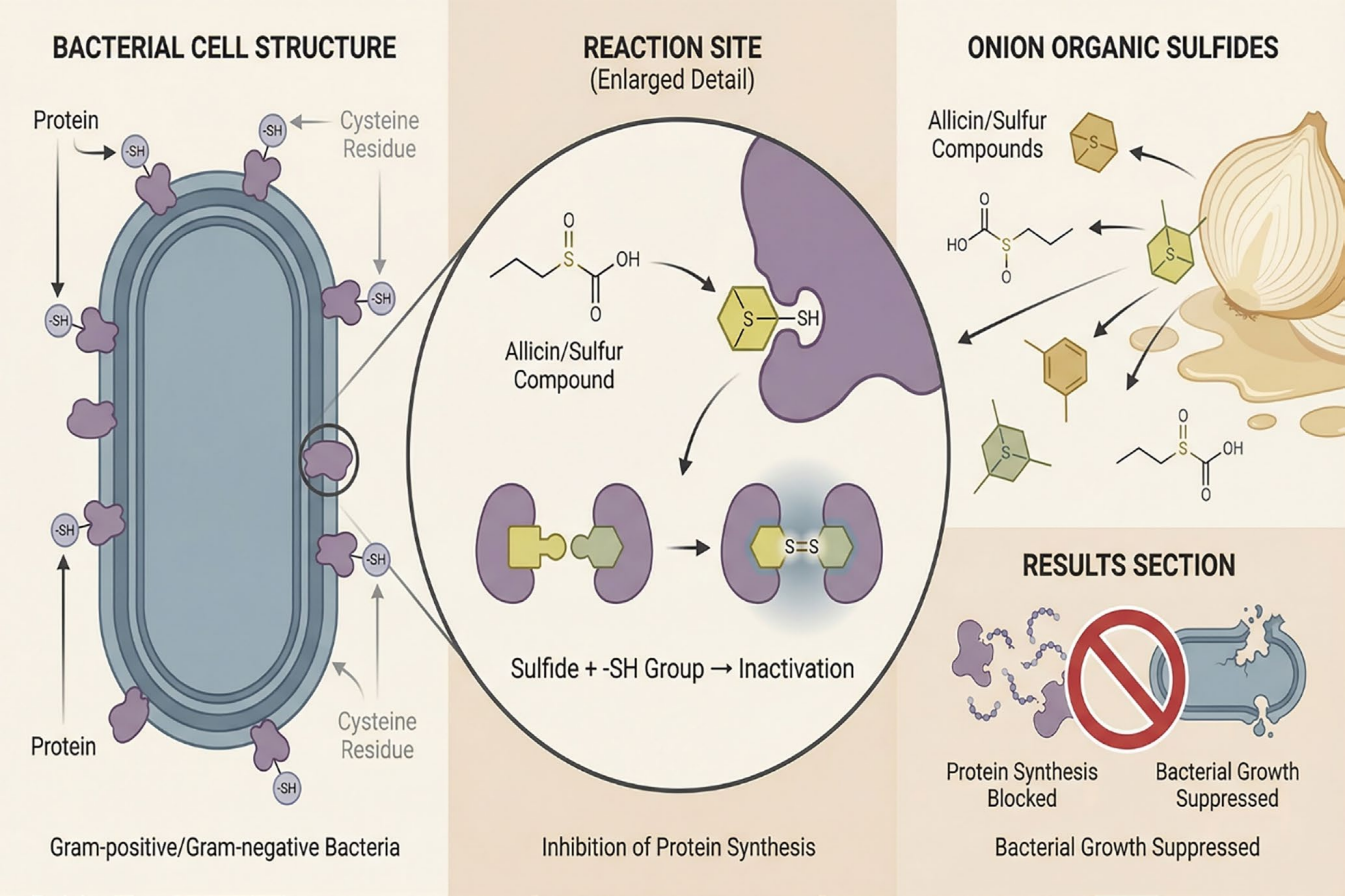
Anti‐bacterial properties of sulfides in onions. Image illustrates the interaction between sulfides in onions and thiol groups (‐SH) present on microbial cell walls. This interaction disrupts the formation of essential proteins, thereby inhibiting microbial growth and reproduction.

Onions' antibacterial properties have been suggested to be due to sulfides reacting with cysteine that have a sulfur group (‐SH) on microorganisms' cell walls, preventing them from forming corresponding proteins and inhibiting their growth and reproduction (Mawouma et al. 2023; Guillamón et al. 2021). Figure 3 demonstrates this process.

### 4.3 Anti‐Tumor Properties of Onion OSCs

According to scientific investigations, the antitumor effects of organic sulfides are primarily attributed to their ability to inhibit carcinogen activation, enhance intracellular detoxification enzyme systems, repair DNA damage, and modulate tumor cell gene expression to induce apoptosis (Adico et al. 2024; Liang et al. 2024). These compounds also exert anticancer activity by arresting the cell cycle, suppressing tumor metastasis, and inhibiting angiogenesis.

**FIGURE 4:**
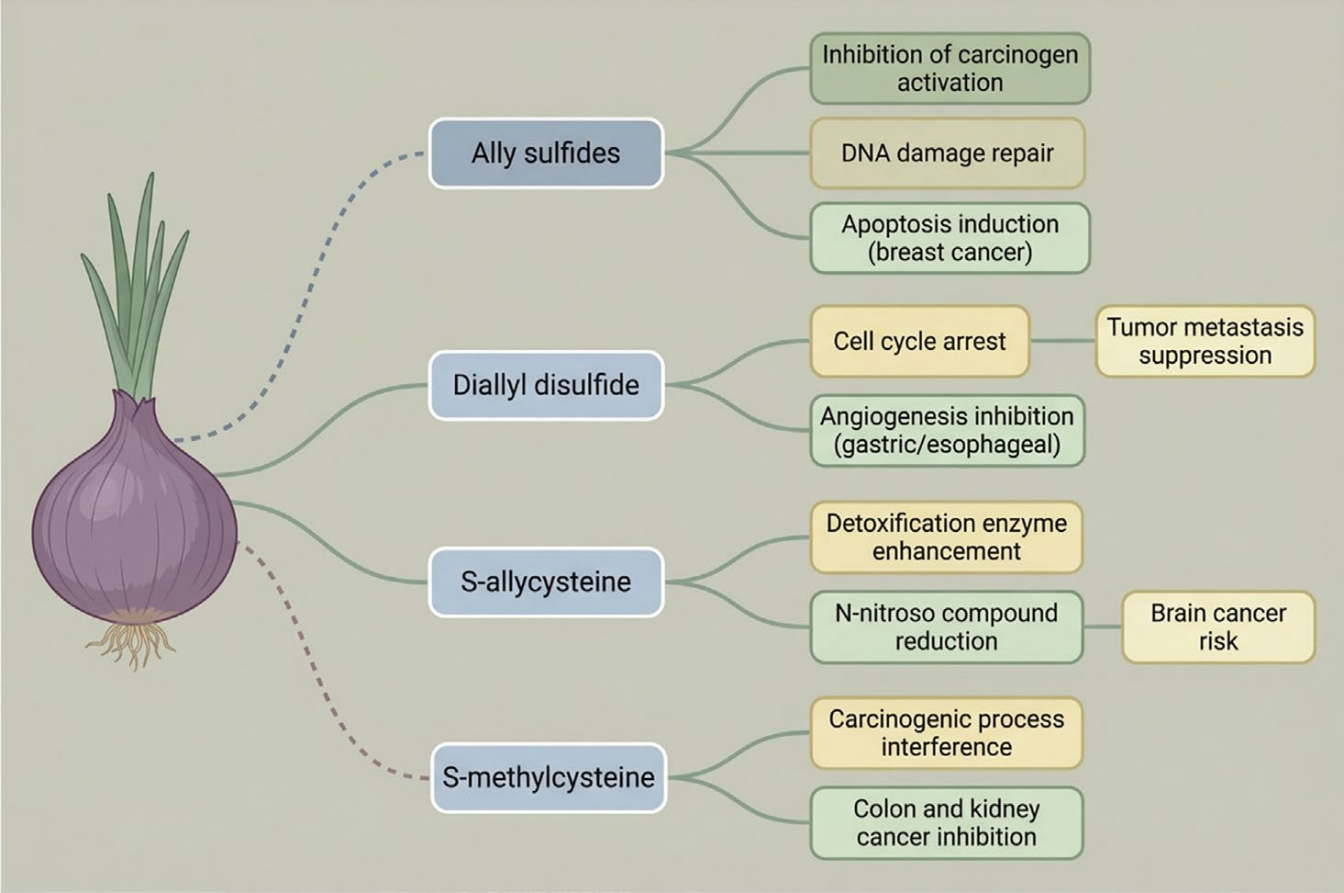
Antitumor properties of sulfides in onions. Image describes the anti tumor mechanisms of major organosulfur compounds produced from onions. The graphic depicts the therapeutic properties of allyl sulfides, diallyl disulfide, S‐allylcysteine, and S‐methylcysteine through multiple pathways, which includes carcinogenesis inhibition, apoptosis induction, cell cycle arrest, metastasis and angiogenesis suppression, and detoxification.

Viry et al. (2011) demonstrated that allyl sulfides found in onions act as preventive agents against breast cancer and exhibit significant inhibitory effects on gastric, esophageal, and colorectal cancers. The mechanism involves inhibition of carcinogen activation, thereby interfering with DNA damage repair and promoting apoptosis induction in breast cancer. Shala et al. (2023) and Sak (2014) further reported that diallyl disulfide, contained in onions, effectively induces cell cycle arrest, suppresses tumor metastasis, and inhibits angiogenesis—particularly in gastric and esophageal cancers. S‐allylcysteine was shown to reduce N‐nitroso compounds derived from pickled foods, thereby lowering the risk of brain cancer (Velmurugan et al. 2005). Furthermore, Ghinet et al. (2016) and Nakajima et al. (2007) indicated that S‐methylcysteine may interfere with carcinogenic processes associated with colon and kidney cancers. Figure 4 illustrates antitumor properties of OSCs derived from onions, particularly Allyl sulfides, Diallyl disulfide, S‐allylcysteine, and S‐methylcysteine.

### 4.4 Onion Compounds in Diabetes Alleviation

**FIGURE 5:**
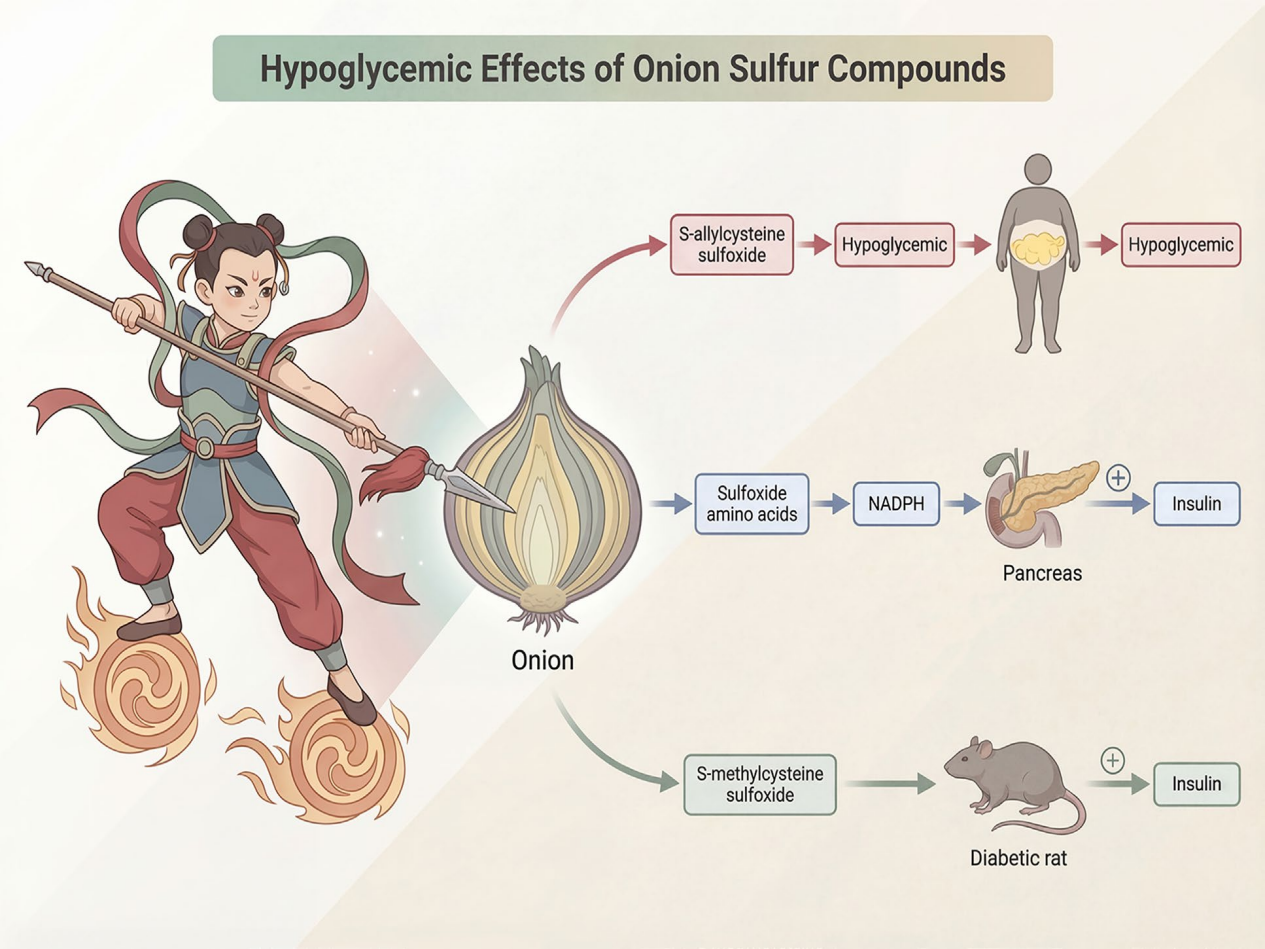
Hypoglycemic effects of onion sulfur compounds. This schematic diagram illustrates the hypoglycemic mechanisms of the primary sulfur‐containing compounds in onions, including S‐allylcysteine sulfoxide, sulfoxide amino acids and S‐methylcysteine sulfoxide. The central focus of the illustration highlights how these bioactive constituents exert hypoglycemic effects through modulation of NADPH levels, insulin secretion, and outcomes observed in diabetic rat models.

Sheela et al. isolated S‐allylcysteine sulfoxide from onions in 1992 and reported that it has hypoglycemic properties (Subramanian et al. 2020). Onions' diabetes‐relieving impact is mainly associated with their extracts' ability to considerably improve indicators such as glucose, urea, creatinine, and bilirubin in the blood (Egbuna et al. 2021; Calis et al. 2020). Investigations have demonstrated that the sulfoxide amino acids identified in onions could interact with NADPH, inhibiting the reduction process of insulin, lowering blood glucose levels, and producing a hypoglycemic impact (Kim et al. 2024). Yang et al. (2018) confirmed, in an experiment on the hypoglycemic effects of garlic oil and onion oil in tetraoxan‐induced diabetic mice, that organic sulfides obtained from onion oil, such as S‐allylcysteine sulfoxide and S‐methylcysteine sulfoxide, are closely associated with diabetic condition improvement. In addition, animal studies have demonstrated that oral administration of sulfur‐containing compounds derived from onions can enhance insulin secretion, modulate hepatic activities of hexokinase and glucose‐6‐phosphatase, and significantly reduce blood glucose levels in alloxan‐induced diabetic rats (Sajitha et al. 2016; Ikechukwu and Ifeanyi 2016). Figure 5 illustrates hypolipidemic effects of onion sulfur compounds.

### 4.5 Anti‐Asthmatic Effects of Onion‐Derived Sulfur Compounds

**FIGURE 6:**
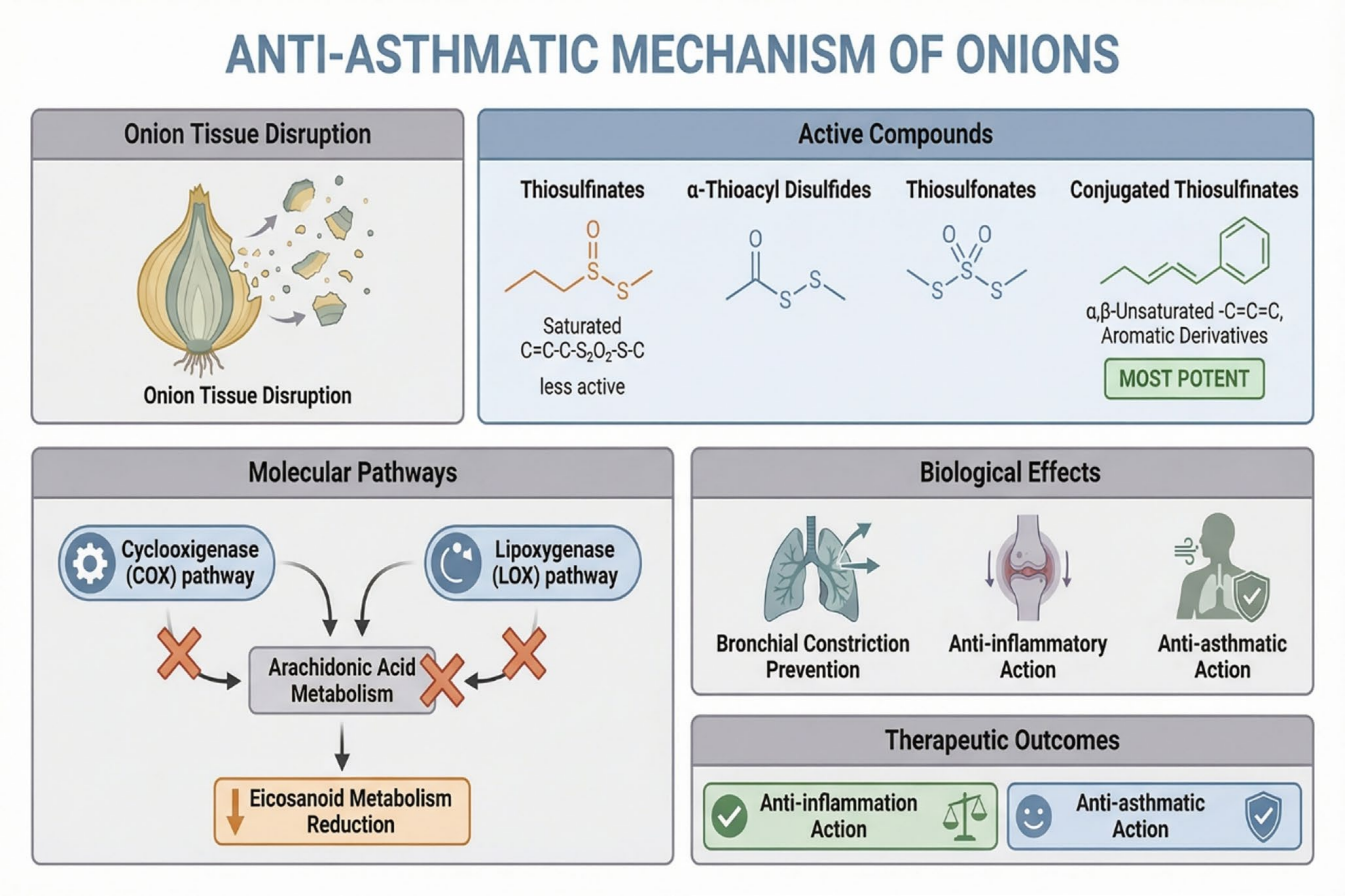
Anti‐asthmatic effects of onion‐derived sulfur compounds. Figure shows onion contain sulfhydryl compounds including thiosulfites, α‐thioacyl disulfides, and thiosulfonates, which significantly influence arachidonic acid metabolism by modulating the cyclooxygenase (COX) and lipoxygenase (LOX) pathways. These components interfere with eicosanoid production, leading to a reduction in eicosanoid metabolism. As a result, they have anti‐inflammatory and asthma‐relieving benefits.

Onions consist of chemical components including thiosulfinates, α‐thioacyl disulfides, and thiosulfonates, which contribute to their anti‐asthmatic effects (Memarzia et al. 2019; Dorsch et al. 1987; Zhao et al. 2021). These sulfur‐containing compounds perform by regulating fundamental biochemical processes involved in inflammation. They significantly impact the metabolism of arachidonic acid, especially the cyclooxygenase (COX) and lipoxygenase (LOX) pathways. Interference with these pathways has important physiologic consequences, particularly a reduction in eicosanoid metabolism. This reduction subsequently helps prevent bronchial constriction (Sharma et al. 2012). Figure 6 demonstrates the mechanisms underlying onion's anti‐asthmatic effectiveness, including molecular structures, biological processes, and therapeutic effects.

Among them, saturated thiosulfinates exhibit lower biological activity than their unsaturated analogs. Conjugated thiosulfinates, particularly α, β‐unsaturated and aromatic derivatives, exhibit the greatest anti‐asthmatic properties (Li et al. 2023, 2017). According to Kianian and Dorsch, the organic sulfur compounds in onions perform an essential part in anti‐asthmatic activity (Kianian et al. 2021; Al‐Sanea et al. 2023; Dorsch et al. 1988). Thiosulfonates, formed during the degradation or mechanical disruption of onion tissues, are capable of effectively suppressing the arachidonic acid metabolic pathway, resulting in both anti‐inflammatory and anti‐asthmatic effects (Yamasaki et al. 2021; Kumar et al. 2015).

In conclusion, Table 2 presents a comprehensive Summary of Key Pharmacological Studies on Onion OSCs, which includes extraction methods, experimental models, doses, key findings, and corresponding references, thereby facilitating a rapid understanding and comparison of onion‐derived sulfur compounds.

**TABLE 2:** Summary of key pharmacological studies on onion organosulfur compounds.

Extract/Compound	Extraction method	Experimental model	Dose/Concentration	Key findings	References.
Onion extract (polyphenol‐rich)	Ethanol extraction	SD rats (hyperlipidemic)	200 mg/kg/day, 4 weeks	Reduced serum TC, TG, LDL‐C; increased HDL‐C; improved lipid profile	Li et al. (2021)
S‐propyl cysteine	Not specified (commercial standard)	HepG2 cells	50–200 μM	Reduced apoB100 secretion and TG synthesis; inhibited lipoprotein assembly	Han et al. (2002)
Cycloalliin	Not specified (isolated compound)	SD rats	100 mg/kg/day, 4 weeks	Reduced serum TG; modulated hepatic lipid synthesis and secretion	Yanagita et al. (2003)
S‐methylcysteine sulfoxide	Not specified (isolated compound)	Alloxan‐induced diabetic mice	50 mg/kg/day, 21 days	Hypoglycemic and hypolipidemic effects; improved duodenal morphology	Castro et al. (2021)
Onion essential oil	Steam distillation	*Salmonella Typhimurium* (in vitro)	0.5–2.0 mg/mL	Exhibited dose‐dependent antibacterial activity; synergistic effect with ultrasound	Kong et al. (2024)
Purple onion fermented extract	Fermentation + solvent extraction	Chickens (in vivo challenge)	10 mL/kg feed, 7 days	Reduced toxin‐carrying bacteria; improved gut health and immunity	Hai et al. (2025)
Allyl sulfides (onion‐derived)	Not specified (commercial)	MCF‐7 breast cancer cells	10–50 μM	Inhibited cell proliferation; induced apoptosis; suppressed carcinogen activation	Viry et al. (2011)
Diallyl disulfide	Not specified (synthetic)	Gastric/esophageal cancer cells	20–100 μM	Induced cell cycle arrest; inhibited metastasis and angiogenesis	Shala et al. (2023); Sak (2014)
S‐allylcysteine sulfoxide	Isolated from onion bulbs	Alloxan‐induced diabetic rats	50 mg/kg/day, 28 days	Enhanced insulin secretion; improved glucose tolerance; regulated hepatic enzymes	Yang et al. (2018)
Onion oil fraction	Solvent fractionation	Lead acetate‐exposed rats	100 mg/kg/day, 30 days	Attenuated oxidative stress and lipid peroxidation; comparable to vitamin E	Sajitha et al. (2016)
Thiosulfinates‐rich extract	Subcritical water extraction	RAW264.7 cells (LPS‐induced inflammation)	10–100 μg/mL	Inhibited COX‐2 and LOX pathways; reduced eicosanoid production; anti‐inflammatory	Trigueros et al. (2024)
Onion‐derived nanoparticles	Aqueous extraction + ultracentrifugation	RAW264.7 cells (LPS‐stimulated)	10–50 μg/mL	Suppressed NO production; anti‐inflammatory effect independent of endocytosis	Yamasaki et al. (2021)

## 5 Conclusion

Onions are widely distributed and have a high production in China. They could be used as both medicine and dietary supplements. Numerous studies have reported the health advantages of organic sulfur compounds found in onions. The academic community has done much research on onion organic sulfur compounds, which exhibit important biological properties. According to the literature, onions and their extracts exhibit an enormous variety of pharmacological effects, including lipid‐lowering, antibacterial, antitumor, antidiabetic, and anti‐asthmatic properties. These numerous biological properties indicate that onion‐based medications might possess potential applications for the treatment of a variety of diseases, especially atherosclerosis, diabetes, tumors, and asthma. However, how to apply the medicinal components of these drugs in clinical practice still requires in‐depth research.

In addition, the current technical challenges involve the inherent pungency of onion‐derived organic sulfur compounds, which restricts their application in highly processed food and pharmaceutical formulations. This limitation underscores the necessity for effective flavor‐masking techniques or structural modification approaches. Future research is expected to uncover additional crucial therapeutic properties by exploring onion organic sulfur compounds in depth, giving onion organic sulfur compounds extensive development and utilization value, as well as the potential to contribute good economic, social, and environmental benefits.

## Author Contributions

**Yijing Tao:** writing – original draft, writing – review and editing. **De Lv:** writing – original draft, writing – review and editing. **Yuanyuan Tang:** investigation, funding acquisition, validation, visualization, project administration.

## Funding

This study was supported by Changshu Health Commission Science and Technology Program of 2022 (CSWSQ202203) and Changshu Science and Technology Program of 2023 (CY202301).

## Ethics Statement

The authors have nothing to report.

## Conflicts of Interest

The authors declare no conflicts of interest.

## Data Availability

The data that support the findings of this study are available from the corresponding author upon reasonable request.
